# Polydextrose-driven gut microbiota modulation from synbiotic yogurt intake simulated in the *in vitro* dynamic multivessel colonic xGI*biomics* system

**DOI:** 10.3389/fnut.2025.1654738

**Published:** 2025-09-04

**Authors:** Rita de Cassia Pessotti, Mateus Salgaço, Laira Lorraine Agostinho, Miller Nunes de Freitas, Cristina Stewart Bittencourt Bogsan, Cristiano Ruch Werneck Guimaraes, Katia Sivieri

**Affiliations:** ^1^Nintx - Next Innovative Therapeutics, São Paulo, Brazil; ^2^Department of Genetics, Beth Israel Deaconess Medical Center, Harvard Medical School, Boston, MA, United States; ^3^Department of Food and Nutrition, School of Pharmaceutical Sciences, São Paulo State University, Araraquara, Brazil; ^4^Department of Biotechnology, University of Araraquara (UNIARA), Araraquara, Brazil

**Keywords:** gut microbiota, polydextrose, BB-12^®^, prebiotic effect, probiotic

## Abstract

**Introduction:**

The gut microbiota plays a pivotal role in maintaining host health and dietary strategies, such as synbiotic interventions, have emerged as promising tools to modulate its composition and metabolic activity. Inulin is a well established prebiotic, but alternative fibers like polydextrose have gained interest due to their distinct fermentation profiles and functional benefits. This study aimed at evaluating whether polydextrose could exert prebiotic effects comparable to inulin when incorporated into a synbiotic yogurt containing *Bifidobacterium animalis* subsp. *lactis* BB-12^®^.

**Methods:**

Using the *in vitro* dynamic multivessel colonic xGI*biomics*^®^ system, we simulated human gut conditions and assessed changes in microbial composition and metabolite production.

**Results:**

Both synbiotic yogurts increased levels of beneficial organic acids, such as propionate and lactate, and decreased ammonium ion concentrations, indicating a shift toward saccharolytic fermentation. The synbiotic formulation incorporating polydextrose also led to increased butyrate production when compared to the yogurt containing only the BB-12^®^ probiotic, and a greater relative abundance of *Bifidobacterium* spp. when compared to the synbiotic formulation incorporating inulin. Additionally, both synbiotic treatments reduced *Klebsiella* spp., a potentially pathogenic genus.

**Discussion:**

These findings highlight polydextrose as a viable and effective alternative to inulin in synbiotic formulations, reinforcing its potential as a functional dietary fiber for gut health modulation.

## Highlights

- Polydextrose demonstrated prebiotic effects comparable to inulin when incorporated into a synbiotic yogurt containing *Bifidobacterium animalis* subsp. *lactis* BB-12^®^, based on dynamic *in vitro* colonic simulation.
- Both synbiotic yogurts increased beneficial metabolites, including propionate and lactate, while significantly reducing ammonium ion levels, suggesting a shift toward saccharolytic fermentation.
- Polydextrose promoted higher relative abundance of *Bifidobacterium spp*. than inulin, highlighting its potential to enhance the persistence of probiotics in the gut.
- The xGI*biomics*^®^ system revealed that microbial diversity remained stable, but beneficial shifts in microbial composition were observed with both synbiotic treatments.
- Polydextrose is proposed as a viable and effective alternative to inulin for synbiotic yogurt formulations aimed at modulating gut microbiota and promoting colonic health.

## 1 Introduction

The intake of prebiotics and probiotics is an effective strategy to restore microbial balance and promote intestinal health (1). According to ISAPP (2016), a prebiotic is “a substrate that is selectively utilized by host microorganisms conferring a health benefit” (2). Gibson et al. (1) define that a compound qualifies as a prebiotic if it: (i) resists digestion in the upper gastrointestinal tract; (ii) is fermented by beneficial colonic microorganisms; and (iii) selectively stimulates bacteria associated with positive health outcomes (3). Polyphenols and certain vitamins can act indirectly as prebiotics by supporting beneficial bacterial growth and a healthy gut environment (4–9).

Well-established prebiotics include fructooligosaccharides (FOS), galactooligosaccharides (GOS), and inulin (3). Inulin, widely used in the functional food industry, modulates the gut microbiota, replaces fat and sugar in foods, and resists digestion, favoring fermentation by *Bifidobacterium* spp. and *Lactobacillus* spp., which produce short-chain fatty acids (SCFAs) such as acetate, propionate, and butyrate (10–14). Benefits include immune modulation (13), anti-inflammatory (15), antidepressant (16), and antioxidant effects (17, 18), as well as improved glycemic control, satiety (19), and lipid profile (20). Polydextrose, a highly branched glucose polymer, is a potential alternative to inulin. It is slowly fermented throughout the colon, supplying substrates even to the distal region and reducing proteolytic fermentation (21). In Brazil, ANVISA requires at least 2.5 g of dietary fiber per serving for intestinal transit claims—easily met with polydextrose (22). It is also approved by the FDA (23) and EU (24). Clinical evidence shows it improves bowel function, increases fecal bulk, reduces transit time (25, 26), produces SCFAs (27, 28), and lowers toxic metabolites from protein fermentation (29), being safe and well-tolerated even at high doses.

Probiotics are live, non-pathogenic microorganisms that, when administered in adequate amounts, confer health benefits by modulating the gut microbiota (30). Initially, it appeared reasonable to define a synbiotic as a combination of a probiotic and a prebiotic, with each component independently meeting the established criteria for probiotics or prebiotics (2, 31) However, further discussion of various scenarios and combinations revealed that this restrictive definition overly emphasizes the individual components, rather than the synergistic interaction between them that characterizes a true synbiotic (6). The development of synbiotic formulations represents a promising approach to human health promotion, as it combines the beneficial effects of both components in a single intervention. Accumulating evidence shows that synbiotics can positively modulate the gut microbiota, enhance immune function, and reduce inflammatory markers, even in healthy individuals (7). Moreover, clinical studies support their efficacy in reducing postoperative infections, hospital stay duration, and antibiotic use, highlighting their potential as adjuvant therapy in clinical settings (8). Within a synbiotic formulation, the prebiotic component plays a crucial role by providing a selective substrate for the growth and activity of probiotic microorganisms, thereby enhancing their survival, colonization, and functional efficacy in the gastrointestinal tract (9).

The probiotic component plays a pivotal role in synbiotic formulations, owing to its well-documented capacity to beneficially modulate the gut microbiota. One of the most extensively studied strains is *Bifidobacterium animalis* subsp. *lactis* BB-12^®^ (BB-12^®^), which is widely incorporated into functional foods and dietary supplements (32). Clinical evidence indicates that BB-12^®^ supplementation improves bowel regularity, increases evacuation frequency, reduces intestinal transit time, and alleviates symptoms such as constipation and abdominal discomfort (33–35). This strain also exhibits immunomodulatory properties, strengthening the intestinal barrier and reducing infection incidence, particularly in vulnerable populations such as children and the elderly (36–38). Its resistance to gastric pH and bile, along with its strong adherence to the intestinal mucosa, enhances its survival and colonization in the gastrointestinal tract (39, 40).

In light of these findings, the present study seeks to determine whether polydextrose can be considered a functional equivalent to inulin—currently regarded as the gold standard among prebiotic compounds for application in synbiotic formulations. To this end, two synbiotic yogurt formulations incorporating *Bifidobacterium animalis* subsp. *lactis* BB-12^®^ were comparatively assessed, differing exclusively in the prebiotic ingredient employed (inulin vs. polydextrose). To simulate human colonic fermentation, the xGI*biomics*^®^ technology was employed, a proprietary *in vitro* dynamic multivessel colonic system (41, 42).

## 2 Materials and methods

A comprehensive list of all reagents and chemicals used in the experiments, including digestion reagents, SCFA standards, and their respective suppliers, has been provided in Supplementary Tables S1–S6.

### 2.1 Collection of fecal material

This study was approved by the Bioethics Committee of Anhanguera University (CAAE 57389722.2.0000.5493) and conducted in accordance with the ethical principles of the Declaration of Helsinki. Eligible participants were healthy adults aged 18 years or older. Individuals were excluded if they had used any medications (e.g., antibiotics, antidiabetics, antihypertensives), consumed probiotics or prebiotics, or experienced illness within 30 days prior to sample collection. Three participants who met these criteria were enrolled. Therefore, fresh human fecal samples from three healthy adult were obtained. To prepare the fecal inoculum, 10 g of feces were homogenized using a stomacher and diluted to 100 ml with 0.1 M phosphate-buffered saline (PBS, pH 7.3). The mixture was centrifuged at 3,000 g for 15 min, the supernatant was homogenized with glycerol (30% v/v) and stored at −80 °C in an ultra-freezer (43). For colonic reactor inoculation, a pooled inoculum of 240 ml was prepared by combining 80 ml from each donor. The pooled sample was homogenized in a sterile flask, and 40 ml of this mixture was used to inoculate the colonic reactors.

### 2.2 Long-duration *in vitro* dynamic multi-vessel colonic simulation using xGI*biomics*^^®^^ technology

The xGI*biomics*^®^ technology (41) is an advanced, *in vitro* dynamic model of the human gastrointestinal tract, operated by dedicated software and consisting of five sequentially connected reactors that mimic the stomach (R1), small intestine (R2), ascending colon (R3), transverse colon (R4), and descending colon (R5). Each compartment replicates the conditions of the respective simulated organ compartment with respect to pH, temperature, retention time, and volume (42). The system comprises two identical and parallel lines (A and B), enabling the simultaneous simulation of distinct experimental conditions by varying parameters, such as microbial communities or nutritional inputs. In this study, both lines were utilized and R4 and R5 were used as biological replicates of the ascending colon (pH 5.6–5.9; mean retention time of 20 h), instead of simulating the transverse and descending colons (44).

Simulated gastric conditions in R1 were achieved with a pH of 2.3–2.5 and a residence time of 2 h. In the small intestine compartment (R2), artificial pancreatic juice (40 ml, composed of 12.5 g/L NaHCO3, 3.6 g/L Oxgall, and 0.9 g/L pancreatin; Sigma-Aldrich, St. Louis, MO, USA) was introduced at a rate of 4 mL/min, with a retention time of 4 h and without pH regulation. Throughout the system, pH levels were automatically controlled via titration with sodium hydroxide (1 M) or hydrochloric acid (1 M), and the temperature was consistently maintained at 37 °C (45, 46). Anaerobiosis in the colon reactors was sustained by daily nitrogen flushing at 200 ml/min for 60 min (47). To initiate colonization, each ascending colon reactor received 500 ml of sterile nutritional medium containing: 1.0 g/L arabinogalactan (Sigma-Aldrich, St. Louis, USA), 2.0 g/L pectin (Sigma-Aldrich, São Paulo, Brazil), 1.0 g/L xylan (Roth, Karlsruhe, Germany), 3.0 g/L starch (Sigma-Aldrich, São Paulo, Brazil), 0.4 g/L glucose (Synth, Diadema, Brazil), 3.0 g/L yeast extract (Sigma-Aldrich, São Paulo, Brazil), 1.0 g/L peptone (Kasvi, Italy), 4.0 g/L mucin (Sigma-Aldrich, São Paulo, Brazil), and 0.5 g/L cysteine (Sigma-Aldrich, São Paulo, Brazil),supplemented with 40 ml of fecal inoculum supernatant (48).

**Figure 1:**
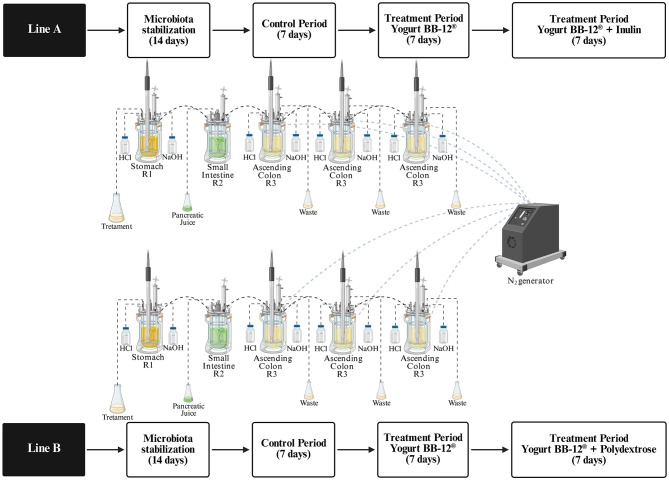
Schematic representation of the experimental protocol for long-term *in vitro* dynamic multivessel colonic simulations using the xGI*biomics* technology.

During the treatment phase, a modified medium with lower carbohydrate content was used, composed of 1.0 g/L arabinogalactan (Sigma-Aldrich, St. Louis, USA), 0.5 g/L pectin (Sigma-Aldrich, São Paulo, Brazil), 1.0 g/L xylan (Roth, Karlsruhe, Germany), 0.4 g/L glucose (Synth, Diadema, Brazil), 3.0 g/L yeast extract (Sigma-Aldrich, São Paulo, Brazil), 1.0 g/L peptone (Kasvi, Italy), 2.0 g/L mucin (Sigma-Aldrich, São Paulo, Brazil), and 0.5 g/L cysteine (Sigma-Aldrich, São Paulo, Brazil) (49). The entire experimental procedure lasted 5 weeks, simulating the gut microbiota of healthy human donors (Figure 1). During the initial 14-day stabilization phase, 300 ml of standard feed medium was added daily to the stomach reactor (R1) of each line, while an equal volume was removed from the ascending colon reactors (R3) every 20 h (45). In the subsequent control phase (7 days), 240 ml of feed and 60 ml of pancreatic juice were administered daily into reactors R1 and R2, respectively.

Following this, two consecutive treatment periods (each lasting 7 days) were implemented. The first treatment for 7 days was identically applied to both lines A and B: 150 ml of yogurt formulated with *Bifidobacterium animalis* subsp. *lactis* BB-12^®^ (DSM 15954) (10^9^ CFU/ml), named Yogurt BB-12^®^, were administered daily, homogenized with 73.2 ml of simulated salivary solution (50), and added with 16.8 ml of pepsin solution (25,000 U/ml; P7000, Sigma-Aldrich, St. Louis, MO, USA). Following this, the second treatment began for additional 7 days, differing between lines A and B. For Line A, inulin was added to Yogurt BB-12^®^ (Yogurt BB-12^®^ + Inulin): 150 ml of synbiotic yogurt formulated with *Bifidobacterium animalis* subsp. *lactis* BB-12^®^ (DSM 15954, 10^9^ CFU/mL) + inulin (2.5 g/100 ml), homogenized with 73.2 ml of simulated salivary solution (50), and added with 16.8 ml of pepsin solution (25,000 U/ml; P7000, Sigma-Aldrich, St. Louis, MO, USA). For Line B, polydextrose was added to Yogurt BB-12^®^ (Yogurt BB-12^®^ + Polydextrose): 150 ml of synbiotic yogurt formulated with *Bifidobacterium animalis* subsp. *lactis* BB-12^®^ (DSM 15954, 10^9^ CFU/ml) + polydextrose (2.5 g/100 ml), homogenized with 73.2 ml of simulated salivary solution (50), and added with 16.8 ml of pepsin solution (25,000 U/ml; P7000, Sigma-Aldrich, St. Louis, MO, USA) (51).

### 2.3 Microbial metabolites analysis

The microbial metabolites, SCFAs, lactate and ammonia, were analyzed from the colonic fermentation samples. Quantification of ammonia was performed in triplicate using a specific ion meter (HI 4101, Hanna Instruments, USA) equipped with an ammonium ion-selective electrode (Orion 95-12, Thermo Fisher Scientific, USA) as previously described by Salgaço et al. (52).

SCFAs, including acetate, propionate, and butyrate, were quantified according to the methodology described by de Oliveira et al. (53). Briefly, colonic fermentation samples were centrifuged at 13,000 g for 5 min, and the resulting supernatants were diluted with Milli-Q water and filtered (0.22 μm PTFE syringe filter) prior to analysis. The samples prepared were then injected into an Agilent gas chromatograph (model HP-6890, Agilent Technologies, Santa Clara, CA, USA), equipped with a selective mass detector (model HP-5975). A DB-WAX capillary column (60 m × 0.25 mm × 0.25 μm) was employed under the following conditions: injector temperature at 220 °C; initial column temperature at 35 °C, ramped at 2 °C/min to 38 °C, then 10 °C/min to 75 °C, 35 °C/min to 120 °C (held 1 min), 10 °C/min to 170 °C (held 2 min), and finally 40 °C/min to 170 °C (held 2 min). The detector was operated at 250 °C, with helium as the carrier gas at a flow rate of 1 ml/min. SCFAs concentrations were determined using calibration curves generated from standard solutions of each acid. All measurements were performed in triplicate, and results are expressed in mmol/L (54).

Lactic acid concentrations were determined using a Shimadzu high-performance liquid chromatography (HPLC) system comprising an LC-10AT pump, Rheodyne injector with a 20 μl loop, CTO-10AS column oven, SCL-10A controller, SPD-M10A diode array detector, and CLASS-VP software. Separation was carried out on a BioRad Aminex HPX-87H column (300 × 7.8 mm), operating in isocratic mode with 2.66 mM sulfuric acid as the mobile phase, at a flow rate of 0.5 mL/min. The column oven was maintained at 65 °C, and detection was performed at 210 nm. Quantification was based on an external standard curve, and data are reported in mmol/L (55).

### 2.4 Prebiotic index (PI) by real-time quantitative PCR (qPCR)

Absolute abundances of total bacteria and specific gut microbial groups were determined by real-time quantitative PCR (qPCR) during the control phase and after 7 and 14 days of treatment, following the methodology described by Mitsou et al. (56). Quantification was performed using SYBR Green I chemistry and genus-specific primers targeting the 16S rRNA gene for *Bifidobacterium* spp., *Lactobacillus* spp., *Bacteroides* spp., *Clostridium* spp., and total bacterial population. Each reaction was carried out in triplicate for every colonic vessel. The primer sequences and reference strains used for calibration are listed in Table 1. To extract microbial DNA, colonic fermentation samples were first centrifuged at 13,000 g for 5 min and the resulting pellets were lyophilized. Lyophilized material was then resuspended in BashingBead Buffer from the Quick-DNA Fungal/Bacterial Miniprep Kit (Zymo Research) and DNA isolation was performed according to the manufacturer's protocol. The purity and concentration of the extracted DNA were assessed using both a NanoDrop spectrophotometer and a Qubit^®^ 3.0 Fluorometer, employing the Qubit™ dsDNA BR Assay Kit (Thermo Fisher Scientific). Quantitative PCR (qPCR) reactions were carried out using a QuantStudio 3 Real-Time PCR System (Thermo Fisher Scientific). Each reaction was performed in triplicate in a total volume of 10 μl, consisting of: 1 μl of forward primer (10 nM), 1 μl of reverse primer (10 nM), 5 μl of PowerUp SYBR Green Master Mix (Cat# A25742, Thermo Fisher Scientific), 1 μl of genomic DNA (gDNA), and nuclease-free water to complete the final volume. Depending on the primer and template concentration, some DNA samples were diluted to optimize amplification efficiency.

**Table 1:** Sequence of primers and reference strains used for quantification of each bacterial group of interest.

**Bacterial Group**	**Primer Sequence 5^′^3^′^, Forward and Reverse**	**Reference Strain**
*Bacteroides* spp.	GAAGGTCCCCCACATTG	*Bacteroides vulgatus* (ATCC 8482)
	CGCKACTTGGCTGGTTCAG	
*Bifidobacterium* spp.	TCGCGTCYGGTGTGAAAG	*Bifidobacterium animalis* subsp. lactis- Bb 12
	CCACATCCAGCRTCCAC	
*Clostridium* spp.	ATGCAAGTCGAGCGATG	*Clostridium perfringens* (ATCC 13124)
	TATGCGGTATTAATCTCCCTTT	
*Lactobacillus* spp.	AGCAGTAGGGAATCTTCCA	*Lactobacillus rhamnosus* (ITAL BL013)
	CACCGCTACACATGGAG	
Total bacteria	TCCTACGGGAGGCAGCAGT	Pool composed by the 4 strains mentioned above
	GGACTACCAGGGTATCTAATCCTGTT	

The thermal cycling protocol included an initial step at 50 °C for 2 min and 95 °C for 2 min, followed by 40 cycles of denaturation at 95 °C for 30 s and annealing/extension at 60 °C for 1 min. Specificity of the PCR products was verified through melting curve analysis conducted immediately after amplification, with fluorescence recorded from 60 °C to 95 °C, increasing by 0.5 °C every 5 s. Quantification of bacterial groups was performed using standard curves generated from DNA of reference strains and results were expressed in log CFU/ml. The PCR efficiency and linearity values for the 5 target genes are shown in Supplementary Table S7. Threshold cycle (Ct) values were automatically calculated using the QuantStudio Design and Analysis Software (Thermo Fisher Scientific).

Samples were taken in triplicate from the colonic vessels during the control phase and after 7 and 14 days of treatment (57). PI was calculated using a formula described by Palframan et al. (58) as follows:


$$\begin{array}{l} Prebiotic\;index\;\left(PI\right)=\\ \left(\frac{Bif}{Total}\right)-\left(\frac{Bac}{Total}\right)+\left(\frac{Lac}{Total}\right)-\left(\frac{Clos}{Total}\right) \end{array}$$

Specifically, “Bif” refers to the numbers of *Bifidobacterium* spp., “Bac” represents the numbers of *Bacteroides* spp., “Lac” denotes the numbers of *Lactobacillus* spp., “Clos” stands for the numbers of *Clostridium* spp., and “Total” indicates the total bacterial numbers. The PI equation assumes a positive effect upon an increase in the populations of bifidobacteria and/or lactobacilli, while an increase in *bacteroides* and *clostridia* (*histolyticum* subgroup) is considered negative. The PI equation provides the advantage of normalizing each bacterial population with respect to the total microbial levels, thereby accounting for the physiological variability inherent in the *in vitro* fermentation (58).

### 2.5 16S rRNA gene amplicon sequencing

The composition of the gut microbial community was assessed through 16S rRNA gene amplicon sequencing, targeting the V4 hypervariable region. The analysis was conducted using the Illumina MiSeq platform (paired-end mode) at BiomaGenetics (São Paulo, Brazil), following their standard protocol.

Genomic DNA was extracted from samples obtained from the long-term *in vitro* dynamic colonic simulation (xGI*biomics*^®^) using the DNeasy PowerSoil Kit (Qiagen), according to the manufacturer's instructions. DNA concentrations were determined using the Qubit Fluorometer (Invitrogen) and the Qubit™ 1x dsDNA HS Assay Kit, followed by normalization to a concentration of 10 ng/μl. Amplicon libraries were constructed using the primer pair 515F-modified (5′-GTGYCAGCMGCCGCGGTAA-3′) and 806R-modified (5′-GGACTACNVGGGTWTCTAAT-3′) (59), which amplify the V4 region of the 16S rRNA gene. PCR amplification was carried out in a total volume of 25 μl containing 12.5 μl of KAPA HiFi HotStart ReadyMix (Roche), 10 ng of DNA template, and 1 μM of each primer. Thermal cycling conditions included an initial denaturation at 95 °C for 3 min, followed by 25 cycles of 30 sec at 95 °C, 30 sec at 58 °C, and 30 sec at 72 °C, with a final extension at 72 °C for 5 min. Following amplification, PCR products were purified using 20 μl of AMPure XP magnetic beads (Beckman Coulter), applying two washing steps with 200 μl of 80% ethanol each, and a final elution step with 52.5 μl of Tris-HCl (pH 8.5), in accordance with the manufacturer's instructions. Unique dual indexes were then ligated to each sample using the Nextera XT Index Kit v2 Set A (Illumina), followed by a second purification step with AMPure XP beads. Post-purification, libraries were re-quantified using the Qubit system and normalized to a final concentration of 4 nM. Equal volumes of each normalized library were pooled to create a combined library with a final concentration of 4 nM. Sequencing was performed using an Illumina MiSeq V2 cartridge (2 × 150 cycles).

### 2.6 Bioinformatics analysis

Sequencing data generated from the Illumina MiSeq platform were processed using the DADA2 pipeline (60), which utilizes a model-based approach to correct sequencing errors and resolve exact amplicon sequence variants (ASVs), allowing fine-scale discrimination of microbial taxa with minimal false positives (61). The full DADA2 workflow was executed using the DADA2 R package (60), encompassing quality filtering, dereplication, error rate learning, sample inference, paired-end read merging, and chimera removal.

Reads were filtered with the filterAndTrim function using the following parameters: trimRight = 0, trimLeft = 0, maxEE = 1.51, truncQ = 2, and maxN = 0, while default settings were used for the remaining arguments. Error rates were estimated iteratively through the learnErrors function (multithread = TRUE, randomize = TRUE, MAX_CONSIST = 50). High-resolution inference of sample composition was carried out using the dada function with multithread = TRUE and pool = TRUE. Denoised paired-end reads were merged with the mergePairs function (minOverlap = 8). ASV tables were generated, and chimeric sequences were identified and removed using a consensus method across samples via the removeBimeraDenovo function (multithread = TRUE). Taxonomic classification of ASVs was performed using the assignTaxonomy function with the SILVA reference database (version 138.1) (62). Further data processing was conducted in the R environment using the MicrobiomeAnalystR package (63). Rarefaction was applied as a normalization strategy to standardize sequencing depth across samples and improve estimates of species richness (64). Diversity analyses were performed on normalized data using the phyloseq package (65) to compute both alpha and beta diversity metrics. Data visualizations were generated with ggplot2 (66).

### 2.7 Statistical Analyses

All analyses were performed in triplicate for each sampled data point per colonic vessel (technical replicates), along with three independent colonic fermentations (biological replicates) using the xGI*biomics*^®^ technology. Results of bacterial metabolites and prebiotic index are presented as means ± standard deviation. The Shapiro–Wilk test was used to assess the normality of bacterial metabolite and qPCR data prior to performing parametric tests. Statistical significance was assessed by Student's *t*-test or one-way analysis of variance (ANOVA) with a significance level set at *p* < 0.05. When applicable, Tukey's multiple comparisons test was conducted as a post hoc analysis. All statistical analyses were performed using GraphPad Prism software, version 8.0 (La Jolla, CA, USA).

## 3 Results

The effects of the consumption of two synbiotic yogurt formulations on the metabolism and composition of the gut microbiota were compared using the xGI*biomics*^®^ technology, a proprietary *in vitro* dynamic colonic fermentation system (41). Both formulations were composed of the BB-12^®^ probiotic (32) and a fiber, either inulin or polydextrose, aiming at investigating how the prebiotic effects of polydextrose compare to those of inulin, a well-stablished prebiotic ingredient in the market (10). The colonic fermentations were conducted using microbiota sampled from healthy adults. The treatments were performed in two steps: first, the microbiota was treated with one portion of yogurt containing only BB-12^®^ (Yogurt BB-12^®^) daily for 7 days, followed by treatments for another 7 days with either one portion daily of the synbiotic yogurt containing BB-12^®^ and inulin (Yogurt BB-12^®^ + Inulin) or one portion daily of the synbiotic yogurt containing BB-12^®^ and polydextrose (Yogurt BB-12^®^ + Polydextrose).

### 3.1. Metabolic activity: SCFAs, lactic acid, and ammonium ions production

The metabolic activity was measured by quantitative analysis of SCFAs (acetic, propionic and butyric acids), lactic acid, and ammonium ions production. When compared to the treatment with Yogurt BB-12^®^, both synbiotic yogurts increased the production of propionic and lactic acids (*p* < 0.05) at similar levels (Tables 2, 3). Yogurt BB-12^®^ + Polydextrose increased butyric acid production (*p* < 0.05), but levels were comparable to the treatment with inulin. Acetic acid production increased upon treatment with Yogurt BB-12^®^ when compared to the control period but remained unchanged during the treatment with the synbiotic yogurts. On the other hand, the ammonium ion production decreased, reaching similar levels by the end of both treatments with synbiotic yogurts (Tables 2, 3).

**Table 2:** Metabolic activity of the microbiota treated with Yogurt BB-12^®^ and synbiotic Yogurt BB-12^®^ + Inulin. Production of short-chain fatty acids (mmol/L), lactate (mmol/L), and ammonium ions (ppm) during the control period and following each treatment, utilizing the *xGIbiomics*^®^ *in vitro* colonic fermentation system and fecal microbiota from healthy adult donors.

**Group**	**Acetic acid**	**Propionic acid**	**Butyric acid**	**Lactic acid**	** ${\text{NH}}_{4}^{+}$ **
Control	32.32 ± 3.10^b^	3.34 ± 2.40^a^	0.20 ± 0.26^a^	10.04 ± 3.70^c^	673.78 ± 19.54^a^
Yogurt BB-12^®^	46.91 ± 6.93^a^	0.00 ± 0.00	0.26 ± 0.05^a^	52.84 ± 3.77^b^	319.11 ± 5.23^b^
Yogurt BB-12^®^+Inulin	44.22 ± 2.28^a^	4.93 ± 3.72^a^	0.15 ± 0.09^a^	79.61 ± 10.27^a^	288.67 ± 6.20^c^

^a, b, c^Different letters indicate a statistical difference in the same column (*p* < 0.05, according to Tukey's post-test).

**Table 3:** Metabolic activity of the microbiota treated with Yogurt BB-12^®^ and synbiotic Yogurt BB-12^®^ + Polydextrose. Production of short-chain fatty acids (mmol/L), lactate (mmol/L), and ammonium ions (ppm) during the control period and following each treatment, utilizing the xGI*biomics*^®^ *in vitro* colonic fermentation system and fecal microbiota from healthy adult donors.

**Group**	**Acetic acid**	**Propionic acid**	**Butyric acid**	**Lactic acid**	**NH${\text{NH}}_{4}^{+}$**
Control	28.58 ± 2.19^b^	0.72 ± 0.55^b^	0.07 ± 0.05^b^	16.17 ± 4.26^c^	661.56 ± 15.38^a^
Yogurt BB-12^®^	41.42 ± 10.33ª	0.54 ± 0.58^b^	0.10 ± 0.03^b^	45.57 ± 6.71^b^	334.00 ± 11.08^b^
Yogurt BB-12^®^ + Polydextrose	41.93 ± 1.79^a^	4.61 ± 2.38^a^	0.29± 0.03^a^	86.02 ± 11.49^a^	234.67 ± 5.83^c^

^a, b, c^Different letters indicate a statistical difference in the same column (*p* < 0.05, according to Tukey's post-test).

The increased production of SCFAs and lactic acid during both synbiotic treatments indicates that the microbiota fermented both fibers, and the fact that the production levels were similar for both suggests that the fermentation occurred at a similar rate. The decreased production of ammonium ions demonstrates that both synbiotic yogurt formulations favored saccharolytic fermentation over proteolytic fermentation. Therefore, in terms of metabolic activity, the synbiotic yogurt containing polydextrose performed similarly to the synbiotic yogurt containing inulin; both displaying beneficial aspects for gut and overall health. These results highlight polydextrose as an alternative for inulin in a synbiotic formulation in terms of production of beneficial microbial metabolites in the gut.

### 3.2. Microbial composition during *in vitro* colonic fermentation and prebiotic effects

#### 3.2.1. Microbial diversity

**Figure 2:**
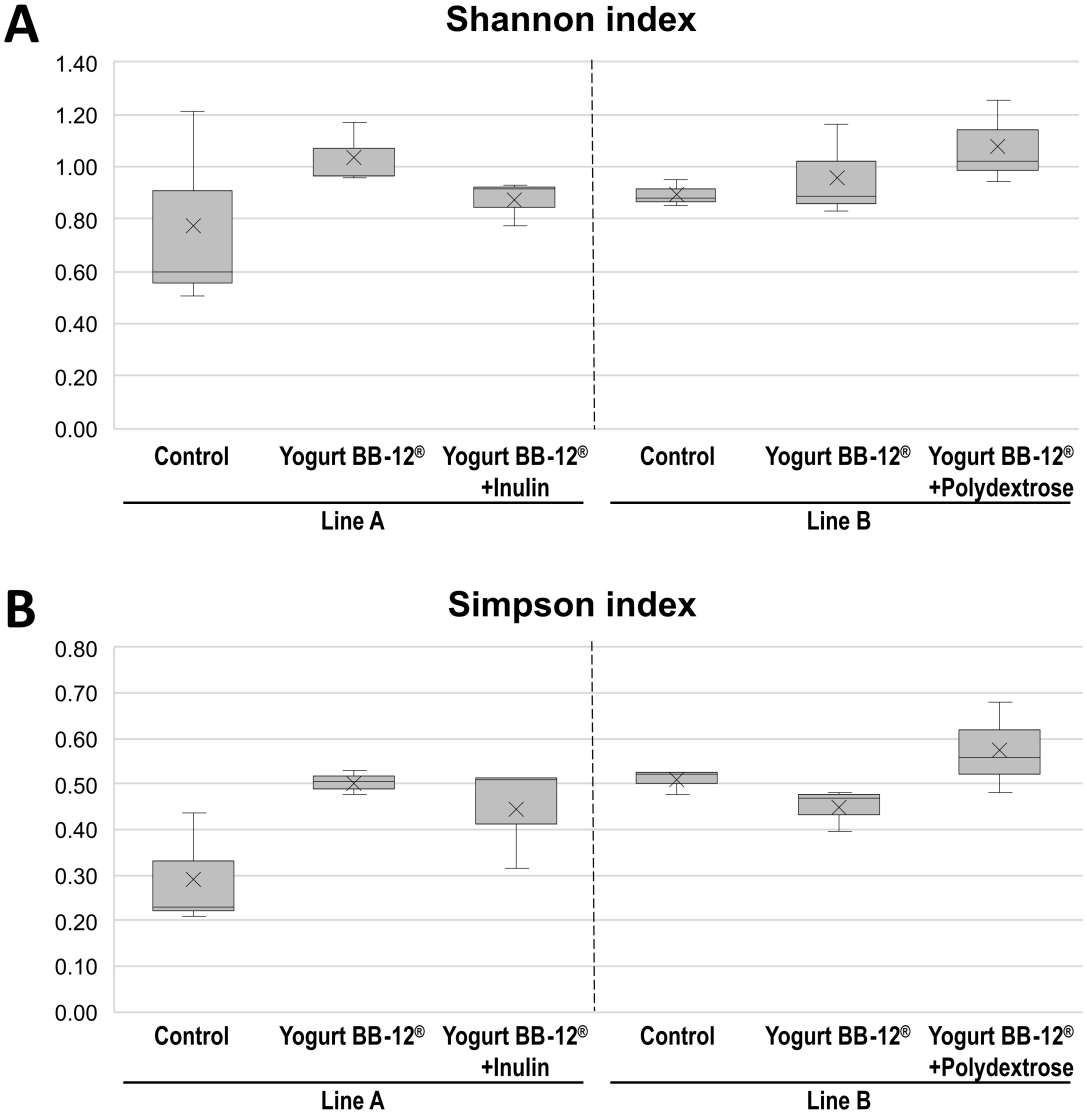
Alpha-diversity of the microbiota treated by Yogurt BB-12^®^ and the synbiotic yogurts. Alpha-diversity (Shannon and Simpson indices, calculated at the family level) of gut microbiota from healthy adults during *in vitro* colonic fermentation utilizing the xGI*biomics* model, before treatments (control) and upon daily administration of the Yogurt BB-12 for 7 days, followed by daily administration of the synbiotic Yogurt BB-12 containing either inulin (line A) or polydextrose (line B) for another 7 days.

**Figure 3:**
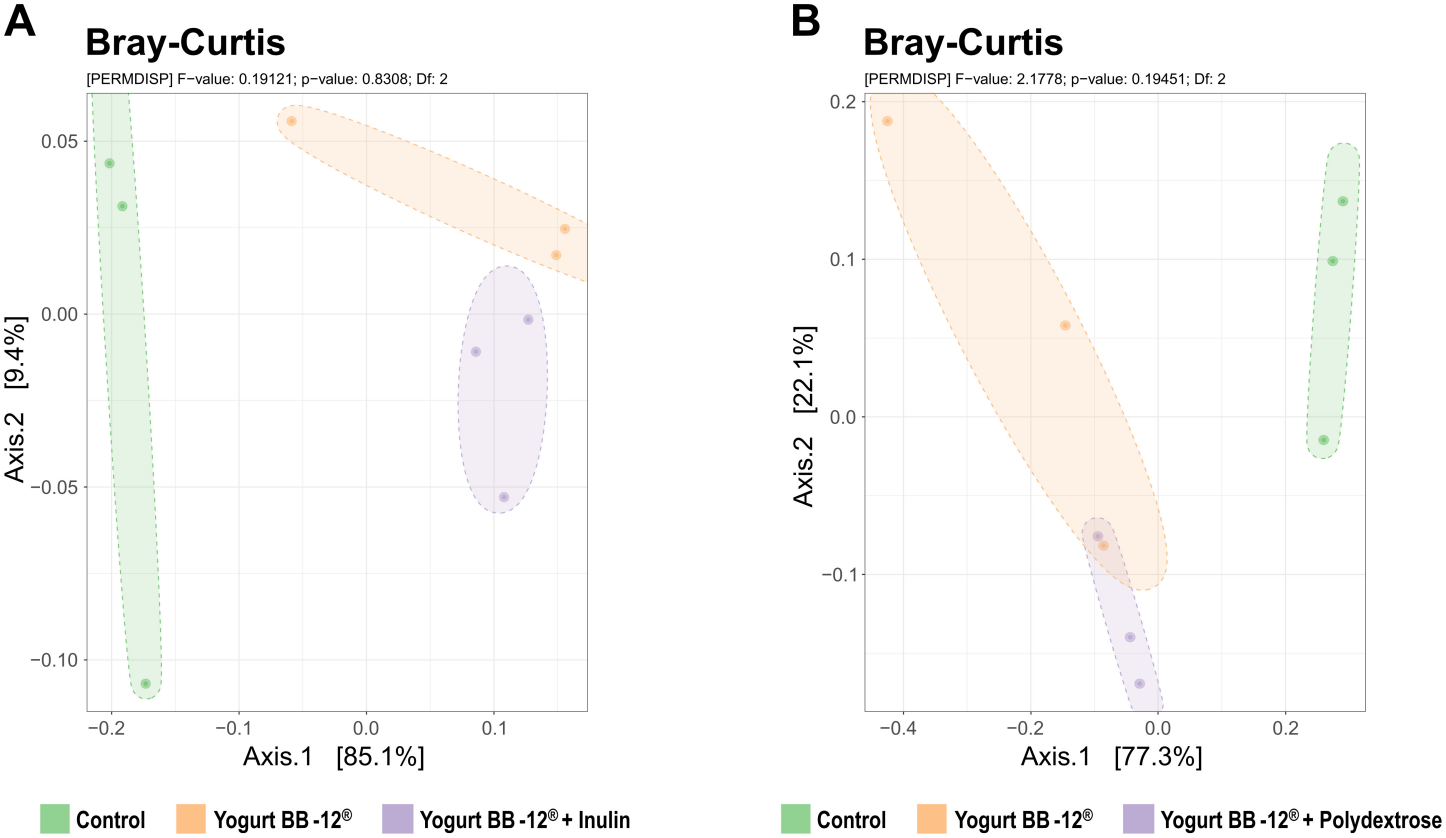
Beta-diversity of the microbiota treated by Yogurt BB-12 and the synbiotic yogurts. Beta-diversity (Bray-Curtis index calculated at family level) of gut microbiota from healthy adults during *in vitro* colonic fermentation utilizing the xGI*biomics* system, before treatments (control) and upon daily administration of the Yogurt BB-12 for 7 days, followed by daily administration of the synbiotic Yogurt BB-12 containing either inulin (line A) or polydextrose (line B) for another 7 days.

In order to analyze the impact of the treatments on the microbial community composition, 16S rRNA gene amplicon sequencing was performed. First, the Shannon and Simpson indices (alpha-diversity) were calculated for each sample (Figure 2). Then, samples within the same fermentation line were compared using beta-diversity (Bray-Curtis index) (Figure 3).

The Shannon and Simpson alpha-diversity and Bray-Curtis beta-diversity indices did not show any statistical difference among the treatments. Most of the differences were observed between the control period and the treatment period. Nonetheless, a trend was observed toward a slightly increased Simpson index upon the administration of Yogurt BB-12^®^ + Polydextrose (on average, 12.5% higher than the control period, Figure 2). Therefore, these results suggest that the overall structure of the microbial community, in terms of richness and evenness, did not go through substantial changes when comparing the synbiotic yogurts, once more substantiating that the synbiotic yogurts with either inulin or polydextrose may have similar impacts on the gut microbiota of healthy adults.

#### 3.2.2. Prebiotic index (PI) and microbial relative abundance

PI was measured by the quantification of selected bacterial genera, followed by the use of a formula described by Palframan et al. (58). Even though PI increased upon treatment with the Yogurt BB-12^®^ in each xGI*biomics*^®^ experimental line, it remained stable after the addition of the synbiotic yogurts (Table 4). Compared to the control period, PI increased by 0.43 and 0.42 upon treatment with the synbiotic yogurts with polydextrose and inulin, respectively. An increase in PI indicates a beneficial shift in the composition of the gut microbiota by increasing the quantity of beneficial genera (here represented by *Lactobacillus* spp. and *Bifidobacterium* spp.) and decreasing the quantity of the usually detrimental genera (here represented by *Clostridium* spp. and *Bacteroides* spp.). Although alpha- and beta-diversity metrics did not indicate overall shifts in diversity, analysis of specific genera within the prebiotic index suggests that Yogurt BB-12^®^ promoted a beneficial modulation of the microbiota, which was sustained by the addition of either fiber.

**Table 4:** Prebiotic index variation during treatments with Yogurt BB-12 and the synbiotic yogurts. Variation of PI during each treatment compared to the control period of each experimental line (Δ Prebiotic index), utilizing the xGI*biomics*^®^ *in vitro* colonic fermentation system and fecal microbiota from healthy adult donors.

**xGI*biomics*^^®^^ line**	**Group**	**Δ Prebiotic index**
Line A	Yogurt BB12^®^	0.43
	Yogurt BB-12^®^ + Polydextrose	0.43
Line B	Yogurt BB-12^®^	0.31
	Yogurt BB12^®^ + Inulin	0.42

**Figure 4:**
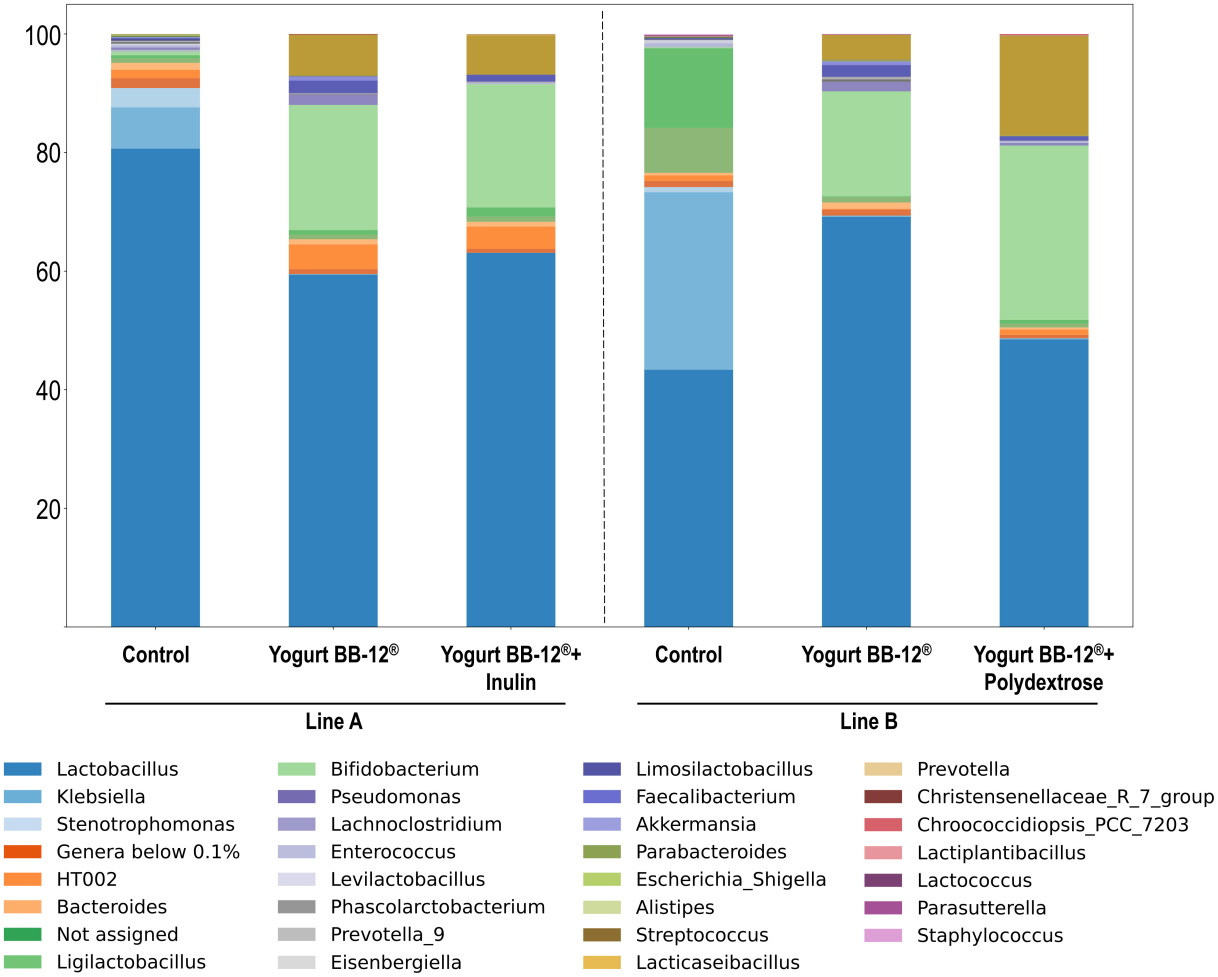
Relative abundance of bacterial genera that compose the microbiota treated by Yogurt BB-12^®^ and the synbiotic yogurts. Composition of gut microbiota from healthy adults during *in vitro* colonic fermentation utilizing the xGI*biomics* system, before treatments (control) and upon daily administration of the probiotic yogurt BB-12^®^ for 7 days, followed by daily administration of the synbiotic yogurt BB-12^®^ containing either inulin (line A) or polydextrose (line B) for another 7 days.

To further assess the microbial shifts induced by the treatments, the relative abundances of the bacterial genera were investigated in each group (Figure 4).

Overall, no major differences were observed between treatments with the synbiotic yogurts with either inulin or polydextrose beyond shifts in the relative abundance of some genera that were already on the top taxa, such as *Lactobacillus* spp., *Bifidobacterium* spp. and *Streptococcus* spp. (Figure 3). This result is consistent with the patterns detected in both alpha- and beta-diversity analyses. Most of the compositional changes were observed when comparing the control period and the subsequent treatments, as also indicated by the diversity metrics. *Bifidobacterium* spp. started as a minor component at the control period: 0.1% and 0.5% in line A and B, respectively, but increased substantially after the treatment with Yogurt BB-12^®^ to 17.7% and 21.1% in lines A and B, respectively, as expected for a probiotic product containing BB-12^®^. Notably, this proportion was maintained in line A after the treatment with Yogurt BB-12^®^ + Inulin (20.8%) and further increased in line B after the treatment with the Yogurt BB-12^®^ + polydextrose (29.4%). Therefore, these results suggest that while both fibers sustained the presence of the probiotic in the gut microbiota, polydextrose provided additional growth when compared to inulin. The same behavior was observed for *Streptococcus* spp. The opportunistic pathogen *Klebsiella* spp. was initially among the most dominant genera in the control period (29.9% in line A and 6.9% in line B), but its abundance sharply decreased following the first treatment (0.2% in line A and 0.1% in line B) and remained low after the second treatment. Lactobacillus spp. remained a dominant genus under all conditions; however, in line B, its relative abundance decreased after the treatment with Yogurt BB-12^®^ + polydextrose, reversing the increase observed after treatment with Yogurt BB-12^®^ It is also worth noting that, despite both synbiotic yogurts promoted the same top three genera (*Lactobacillus* spp., *Bifidobacterium* spp. and *Streptococcus* spp.), the one with polydextrose promoted a more even distribution among these three, which could account for the observed slightly higher Simpson index.

Altogether, in terms of microbial composition, the synbiotic yogurt containing polydextrose performed similarly to the synbiotic yogurt containing inulin, in accordance with their similar contributions to the metabolic activity. Both synbiotic yogurts presented beneficial microbial shifts, with polydextrose presenting important aspects such as better balancing among the predominant genera, and slightly higher proportion of *Bifidobacterium* spp., highlighting polydextrose as an interesting alternative for inulin in a synbiotic yogurt formulation.

## 4 Discussion

This study aimed at comparing the prebiotic effects of two synbiotic yogurt formulations containing *Bifidobacterium animalis* subsp. *lactis* BB-12^®^ in combination with either inulin or polydextrose. The use of the *in vitro* dynamic colonic fermentation xGI*biomics*^®^ system enabled the simulation of human gastrointestinal conditions and allowed for a detailed analysis of microbial composition and metabolic activity over time. Our findings suggest that polydextrose can exert prebiotic effects comparable to inulin, including modulation of gut microbiota and enhanced production of beneficial fermentation metabolites.

Both synbiotic formulations increased levels of propionate and lactate, while reducing ammonium ion concentrations, indicating a shift toward saccharolytic fermentation. Notably, butyrate and propionate production were significantly enhanced in the polydextrose group and did not differ from that observed in the inulin group. These findings are supported by *in vitro* studies showing that polydextrose significantly increased the production of propionate and butyrate compared to an equivalent dose of inulin, their blends (67) and soluble resistant glucans (68).

Polydextrose is a synthetic glucose polymer with a highly branched structure that resists digestion in the small intestine and undergoes gradual fermentation along the entire colon. This slow fermentability contributes to its high gastrointestinal tolerance and enables prolonged SCFA production, even in distal colonic regions (69). Furthermore, by decreasing ammonium ion levels, polydextrose may reduce the generation of potentially harmful nitrogenous metabolites, contributing to a healthier colonic environment (21). In a batch colonic fermentation model, polydextrose showed after 24 h of fermentation an increase in gas and ammonia production (67). However, our results demonstrated a decrease in ammonia ions in both synbiotic yogurts, indicating that the dairy matrix, as well as the probiotic strain, may mitigate the production of uremic toxins, including ammonia, which can exacerbate conditions such as chronic kidney disease (70).

SCFAs, particularly butyrate, play crucial roles in host physiology and in maintaining gut barrier integrity (71). Butyrate helps regulate metabolism, facilitates fluid transport across the intestinal epithelium, reduces inflammation, and enhances the epithelial defense barrier (72). Studies show that butyrate supplementation improves intestinal health and growth performance in livestock by enhancing intestinal development, improving immune function, and modulating microbial diversity (73). Furthermore, butyrate producing bacteria metabolizes oligosaccharides, which are derived from the host diet and glycans originating from host mucus. Another important finding was the increase in propionate following treatment with synbiotic yogurts, which plays a key role in maintaining gut homeostasis. SCFAs like propionate lower the pH of the colonic lumen, enhancing mineral absorption and inhibiting the growth of pathogenic bacteria (74). Additionally, propionate can modulate the gut microbiota composition (75), strengthen the intestinal barrier and reduce bacterial translocation and systemic inflammation (76).

The administration of synbiotic yogurts significantly influenced specific aspects of microbial composition, even though overall diversity measures remained unchanged. Both alpha-diversity (Shannon and Simpson indices) and beta-diversity (Bray-Curtis dissimilarity) metrics did not exhibit statistically significant differences between treatment groups. This finding is consistent with a systematic review and meta-analysis that assessed the impact of dietary fiber interventions on gut microbiota composition in healthy individuals. Across studies lasting from 4 days to 3 weeks, even substantial increases in fiber intake did not result in alterations in microbial diversity (77). These results suggest that short-term dietary interventions may be insufficient to induce measurable changes in alpha-diversity. In addition, dairy synbiotic interventions may not drastically alter global microbial diversity in healthy individuals (78). However, it is important to note that the effect of polydextrose or inulin on alpha-diversity can depend on various factors, including dosage, duration of intervention, the host's pre-existing gut microbiota composition, and the presence of other dietary components (79).

Nevertheless, genus-level analyses revealed notable microbial modulations. The probiotic yogurt containing *Bifidobacterium animalis* subsp. *lactis* BB-12^®^ led to a marked increase in the relative abundance of *Bifidobacterium* spp., indicating successful colonization and metabolic activity of the probiotic strain. This effect was maintained following both synbiotic treatments, but most pronounced in the group receiving BB-12^®^ combined with polydextrose (29.4%), compared to the inulin group (20.8%). These results align with prior studies reporting that polydextrose selectively promotes bifidobacterial growth and contributes to the persistence of probiotic strains during gastrointestinal transit (80). In addition to supporting *Bifidobacterium* spp., the synbiotic yogurts sustained the dominance of *Lactobacillus* spp. and *Streptococcus* spp., genera often associated with host health and mucosal barrier protection (81). Interestingly, polydextrose appeared to enhance a more even distribution among these top genera, potentially reflecting improved ecological balance and niche sharing. This compositional evenness may explain the slightly higher Simpson index observed in the polydextrose group, which is indicative of community stability (82). Importantly, both yogurt treatments led to a sharp decrease in the abundance of *Klebsiella* spp., a genus associated with opportunistic infections and inflammation (83). This decline, observed after probiotic yogurt administration and sustained by the synbiotic formulations, suggests a potential protective effect through competitive exclusion or environmental acidification driven by SCFA production. While *Lactobacillus* spp. remained prevalent across treatments, its decrease following polydextrose supplementation may reflect interspecies competition or metabolic shifts in substrate preference, a dynamic previously noted in fiber fermentation studies (79).

Collectively, these results indicate that both synbiotic yogurts promoted beneficial microbial shifts by enhancing key health-associated genera while suppressing potentially harmful taxa. Among them, polydextrose demonstrated a slightly superior ability to support bifidobacterial growth and maintain microbial balance, reinforcing its potential as an effective alternative to inulin in synbiotic food formulations (84).

A major strength of this study lies in the use of a dynamic multivessel colonic system that reproduces regional differences along the colon and allows real-time assessment of microbial and metabolic changes. The controlled comparison between two fibers under standardized conditions provides robust evidence of their functional equivalence. However, it is important to acknowledge the limitations of *in vitro* models. Despite their physiological relevance, these systems cannot fully replicate host–microbiome interactions such as epithelial signaling, immune modulation, and systemic metabolite absorption. Moreover, the inter-individual variability of donor microbiota may influence specific responses to dietary fibers. Further clinical studies are needed to confirm the translational relevance of these *in vitro* findings. Longitudinal trials should assess the impact of polydextrose-containing synbiotics on gut health biomarkers, intestinal permeability, systemic inflammation, and host metabolism. Additionally, future research should explore the synergy between polydextrose and different probiotic strains and investigate the long-term resilience and stability of the gut microbiota in response to such interventions.

## 5 Conclusions

This study provides evidence that polydextrose, when incorporated into synbiotic yogurt formulations, exerts prebiotic effects comparable to those of inulin, not only sustaining the growth of *Bifidobacterium animalis* subsp. *lactis* BB-12^®^, but also promoting favorable shifts in key microbial taxa and enhancing the production of health-relevant short-chain fatty acids such as propionate and butyrate. The observed reduction in ammonia ion concentrations suggests that the dairy matrix and probiotic strain can modulate nitrogen metabolism and potentially attenuate the formation of uremic toxins. Importantly, polydextrose displayed a slightly superior capacity to support bifidobacterial abundance and promote compositional balance among dominant gut microbes, reinforcing its value as an effective and functionally versatile dietary fiber. These findings underscore the potential of polydextrose-containing synbiotic formulations as targeted nutritional strategies to modulate the gut microbiota and promote colonic health. Future clinical studies are warranted to validate these effects *in vivo* and explore long-term outcomes related to host metabolic and inflammatory responses.

## Data Availability

The raw data supporting the conclusions of this article will be made available by the authors, without undue reservation.
